# The Spine is the Tree of Life: A Systematic Review and Meta-Analysis of the Radiographic Findings Related to Spinal Injuries in Athletes

**DOI:** 10.7759/cureus.58780

**Published:** 2024-04-22

**Authors:** Chukwuyem Ekhator, Sophia B Bellegarde, Basil N Nduma, Muhammad Qasim Qureshi, Ekokobe Fonkem

**Affiliations:** 1 Neuro-Oncology, New York Institute of Technology, College of Osteopathic Medicine, Old Westbury, USA; 2 Pathology and Laboratory Medicine, American University of Antigua, St. John's, ATG; 3 Internal Medicine, Medical City Hospital, Denton, USA; 4 Medicine, Mayo Hospital, Lahore, PAK; 5 Neuro-Oncology, Barrow Neurological Institute, Phoenix, USA

**Keywords:** spine, athletes, spinal cord injury, flexion, hyperextension

## Abstract

This review article explores spinal injuries in athletes participating in various sporting activities. It also highlights the various mechanisms of injuries that contribute to spinal injuries in each sport. Electronic databases such as PubMed, Cochrane Library, Web of Science, Embase, MEDLINE Ovid, and Google Scholar were searched for articles from 2000 to 2022 on spine injuries in sports and radiological studies discussing the various injury patterns among athletes. Studies were scoured in accordance with the inclusion criteria, and relevant data such as the number of participants, sporting activities, spine injuries, and outcomes were retrieved. Fifteen articles that met the inclusion criteria were included in the study.

Cervical spine injuries are common in athletes who participate in contact sports such as football. Similarly, athletes in collision sports such as football, rugby, and hockey are likely to suffer stingers due to traction and compression injuries. Players engaged in such as soccer, baseball, and swimming, are likely to suffer from spondylolysis. Soccer players are more prone to multiple lesions compared to athletes in sports such as baseball because the sport involves training exercises such as jogging and running without kicking any ball. In swimmers, spondylolysis is common in breaststroke and butterfly styles since they involve repeated flexion and hyperextension of the lumbar spine. CT is essential for diagnosing spondylolysis as it demonstrates the lesions more accurately. Ice hockey is associated with a significant incidence of cervical spine injuries, mostly due to players being constantly checked/pushed from behind. Spine injuries are common in elite athletes across several sports. About 10% of spinal injuries in the United States result from sports activities. In diagnosing spine injuries, imaging modalities such as MRI, CT, or plain radiographs are essential. From a radiologist's perspective, these tests help immensely in deciding which treatment is required for a particular athlete or how the injury can be optimally managed. Achieving recovery from a specific spine injury usually depends on the kind of injury and the rehabilitation process the athletes undergo before returning to play.

## Introduction and background

According to the World Health Organization (WHO), between 250,000 and 500,000 people suffer spine injuries every year globally [1]. Sharma et al. have stated that spinal cord injuries are usually associated with damage to the spinal cord [1]. These injuries constitute extremely serious physical trauma with a potentially lasting and significant impact on various aspects of everyday life [2]. The spinal cord is responsible for sending messages from the brain to all body parts and vice versa, thereby enabling individuals to perceive pain and move their limbs. Therefore, injuries sustained on the spinal cord can lead to “blockage” of some or all impulses, leading to a complete loss of sensation and mobility below the site of injury [3]. As explained by Eldahan et al., spinal cord injuries closer to the neck typically cause paralysis on a broader part of the body than injuries on the lower back area [4].

The most common causes of spinal cord injuries include motor vehicle accidents, falls, acts of violence such as gunshots, medical/surgical complications, and sports or recreational activities [5]. According to a study by the National Spinal Cord Injury Statistical Centre, spanning the period from 2015 to 2019, sporting or recreational activities rank fourth in the most common causes of spinal cord injuries and account for 8% of the total injuries (behind motor vehicle accidents, which account for 39.3%, falls accounting for 31.8%, and violence, which accounts for 13.5%) [3]. Unlike the other causes of spinal injuries, sport-related spine injuries usually occur in young individuals, at a mean age of 24 years [6]. Spine-related injuries are common in all kinds of sports, whether contact or non-contact and at every level starting from high school to professional level [7]. Some of the sporting activities that have been associated with putting participants at a high risk of spinal cord injuries include American football, ice hockey, rugby, skiing, snowboarding, wrestling, basketball, and soccer [8]. Chan et al. have reported that collision sports, especially football, account for most of the spinal injuries [8]. Csobonyeiova et al., Goldberg and Kershah, and Guarnieri et al. suggest that radiographical tests are essential to diagnose and manage these spine injuries [9-11], such as X-rays, CT scans, and MRI.

X-rays are usually important in diagnosing spine injuries because they can reveal vertebral problems, tumors, fractures, or degenerative changes in the spine [12]. On the other hand, Holmes and Akkinepalli et al. state that CT scans offer a clearer image of abnormalities observed in X-rays [12]. According to Okunlola et al., a CT test is better than X-rays because it uses computers to form a series of cross-sectional images that can define bone, disc, and other spine-related problems [13]. However, despite a CT scan being a better diagnostic test, MRI has proved to be better in visualizing the spinal cord and identifying herniated discs, blood clots, and other masses that might compress the spinal cord [14]. As per Bozzo et al. and Masci et al., MRI is highly regarded as an alternative to CT and SPECT because of its non-ionizing properties [14,15]. Additionally, MRI is essential for the identification of pathologies related to low-back pain, especially for locating soft tissue damage [16].

The occurrence of spinal injuries in sports is rare. However, Boden and Jarvis highlight that the most common spinal injuries associated with sporting activities include sprain or strain of the cervical, thoracic, or lumbar spine, interventional disc herniation, spinal fractures (spinous process, transverse process, vertebral body, facet joint, and pedicle), and pars defects (spondylolysis and spondylolisthesis) [17]. Spondylolysis and spondylolisthesis are lumbar spine injuries that occur in young athletes who participate in sports involving repetitive hyperextension and rotation across the lumbar spine [18]. The most common athletes associated with these injuries are gymnasts, football players, especially offensive players, and defensive linemen because their sports involve a tremendous degree of hyperextension and vertical loading [17].

Previous studies have evaluated this kind of spinal injury in young athletes; e.g., according to a study by Cyron et al., about 40% of athletes with back pain lasting for more than three months were diagnosed with abnormalities of the pars interarticularis in the lumbar spine [19]. Similarly, a study by Jackson et al. showed that gymnasts have an 11% incidence of spondylotic defects [20]. Another study reported that up to 15% of college football players may have spondylolysis [21]. This kind of lumbar spine injury is more common in adolescent athletes compared to adults [22]. A previous study by Muschik et al. reported that children between 9 and 15 years old are at a higher risk of progressive slippage [23]. The symptoms associated with this spine injury are low-back pain exacerbated by extension and usually without radiculopathy [24]. For a severe slippage, palpation can be used for diagnosis. However, for a less severe slippage, a physical examination can be used to reveal a lumbar muscle spasm. Imaging tests such as plain X-ray films, CT, and bone scanning modalities such as SPECT are used to diagnose lumbar hyperextension [25]. Plain X-ray films can ascertain the degree of slippage if any, while CT is the preferred imaging modality in defining the bone architecture of the pars. In cases of X-ray failure to detect occult and acute “stress,” SPECT scanning is usually used [26].

Various methods are usually employed in the management of par defects. The main goals of par defect management in athletes are to alleviate pain and prevent progression and instability. Non-surgical par defect management is usually dependent on the amount of slippage [27,28]. According to a previous study by Harvey and Tanner, for patients with low-grade slippage, it is recommended that sporting activities are restricted for some time until the pain subsides, after which the patient can gradually resume activities [29]. In the event of pain resumption, it is recommended that lordotic bracing be used for three to six months until the pain subsides [30,31]. As this management process is underway, X-ray films are important as they can show the healing of this defect. In cases where X-ray films give ambiguous results, SPECT scanning is used to assess the degree of healing. Athletes with low-grade slips usually resume their activities only after a very aggressive rehabilitation program.

Another lumbar spine sport-related injury is herniated discs. Athletes in weightlifting and contact sports such as football, and bowling are usually at a higher risk of lumbar disc herniation because these sports are highly associated with axial loading, flexion, and rotation [32]. In most cases, the signs and symptoms of herniated discs begin during weight training or during movements involving pivoting and turning. In some individuals, the symptoms are usually insidious, probably due to the accumulation of minor injuries [33]. Herniated disc injury is more common in older athletes as opposed to adolescent athletes [34]. During the diagnosis of herniated discs in adolescent athletes, physical examination only reveals mild scoliosis or unilateral hamstring tightness. Therefore, for younger patients with persistent herniated disc symptoms, imaging studies are very important and should be considered. The imaging studies involve radiographic tools such as anteroposterior and lateral X-ray films with oblique views for visualization of pars interarticularis and lateral integrity of the spinal alignment [35]. On the other hand, MRI studies are used to delineate the anatomy of the disc and its relation to nerve roots [33]. These tests are essential in making treatment decisions.

Additionally, athletes who engage in high-collision sports such as auto racing and skiing may suffer from major fractures that cause spinal instability. For athletes in other contact sports, minor fractures such as fractures of the transverse process, spinous process, facets, vertebral bodies, and endplates are at times observed [36]. These minor fractures occur due to athletes being engaged in direct blows, forceful rotation, flexion, and compression. The most common symptom of this injury is back pain immediately after the injury onset. MRI is usually used to diagnose these fractures in patients [14]. The management of minor spinal fractures is done conservatively because only one of the spine columns is injured, and hence spinal stability is not threatened. Athletes who have suffered minor fractures such as fractures of the transverse or spinous process are usually allowed to resume full activity once the symptoms have subsided fully, and full range of motion (ROM) has been restored [37].

On the other hand, weightlifters often suffer from mild compression fractures in the anterior vertebra. A study by Stinson et al. reported that exercises such as squats and military press, which involve repetitive flexion and compression of the lumbar vertebral body, are likely to lead to endplate fracture, disc space collapse, or mild body fracture [38]. This kind of spine injury requires the athlete to restrict these activities after recovery to reduce the risk of reoccurrence. Connolly et al. state that facet joint fractures have recently been recognized in sports medicine and are more common than originally recognized [36]. The most common symptoms of facet fracture in athletes are unilateral pain and pain on extension [39,40]. In young athletes, radicular symptoms may manifest an associated epidural hematoma, presumably caused by bleeding in the fracture site [36]. For the efficient diagnosis and monitoring of the facet fracture, axial MR images are usually used. Similar to all the other lumbar spine injuries, the athlete is only allowed to return to play after the symptoms and compressive radiographic abnormalities are resolved.

Besides lumbar spine injuries, athletes are also vulnerable to cervical spine injuries. According to Morrissette et al., ice hockey is considered to have one of the highest cervical spine injury rates [41]. Most cervical spine injuries associated with this sport occur in the C5-7 levels due to the body checking while the head is tilted downwards [42,43]. In the 1980s, the incidence of spine injuries in ice hockey increased tremendously due to the increasing acceptance of checking as part of the game. This increase in spine injuries prompted the International Hockey Federation to penalize checking or pushing from behind in 1994 [44], which led to spine injuries decreasing significantly [45]. Additionally, other changes such as padded boards are being evaluated to further decrease spine injuries associated with the sport. On the other hand, wrestling has been associated with the highest catastrophic injury rates to the cervical spine [43]. The position most frequently associated with a spinal injury is the defensive posture during the takedown maneuver, followed by the down position (kneeling), and the lying position [46]. To prevent these injuries, coaches and referees are encouraged to discourage takedowns and ‘slams’ with excessive force.

Some of the most common cervical spine injuries in athletes are paraspinal muscle strain and cervical ligament sprain. Muscle strains are associated with direct blows or rapid eccentric muscle contraction, while ligament sprains are caused by forced flexion of the head and neck. According to Connolly et al., the symptom that prompts a diagnosis of cervical strain in athletes is acute pain after a contact injury [36]. Initially, anteroposterior, lateral, and odontoid radiographs are obtained, while lateral flexion/extension is obtained to assess the degree of instability. For the management and treatment of the cervical strain, the athlete is subjected to immobilization and receives anti-inflammatories until the pain subsides [47]. Additionally, athletes are expected to demonstrate a painless ROM once the collar is discontinued before they can resume their sporting activities.

Burner syndrome is another cervical spine injury experienced by young athletes [36]. This condition mostly occurs in the C5 and C6 distribution and is marked by temporary burning and weakness. In younger athletes, this injury is usually linked to the brachial plexus. However, the condition is also experienced by adult athletes due to compression of the upper cervical roots. As per Eldahan et al. (2020), when the cervical spine is forced into hyperextension or combined with lateral flexion or shoulder elevation on the affected area, the cervical foramina is narrowed, resulting in transient radiculopathy [4]. The most commonly experienced symptom of this injury is transient paralysis with a burning sensation that runs from the shoulder through the fingertips [48]. The recovery from these injuries occurs normally within 10 minutes. However, for the athlete to be allowed back to play, the symptoms should fully disappear and the cervical assessed to be normal.

Play restrictions are usually placed on the athletes if they experience more than three episodes, have cervical stiffness and tenderness, and have persistent weakness or upper extremities. Bozzo et al. recommend that these athletes undergo MR imaging [14]. The imaging and examination are highly valuable as they can rule out potential anatomical variations or pathology that may pose an increased chance of permanent injury. If there is no risk of permanent damage, the athlete is usually required to undergo a period of rest and upper extremity strength rehabilitation [49]. Athletes are also faced with acute disk herniation, which results from axial loading that increases intradiscal pressure rapidly. Jones and Price highlight that the result of this injury can either be permanent or transient [50]. The symptoms associated with the injury include paralysis of all four extremities, loss of pain and temperature sensation, posterior neck pain, and/or paraspinal spasm [51]. MRI is the gold standard method for diagnosing a herniated disc [52].

As per Harrop et al., athletes are also likely to suffer from catastrophic injuries due to congenital anomalies that change the cervical spine’s structural integrity [53]. One such anomaly is the Klippel-Feil syndrome, which is a condition that involves the failure of segmentation characterized by a fusion of two or more vertebrae [54]. In such a condition, a spear tackler’s spine injury may be induced in collision sports as the number of fused segments increases. Similarly, athletes with Down syndrome are more likely to suffer catastrophic injuries [55]. Athletes with this condition engaging in contact sports and gymnastics are required to be screened using lateral flexion-extension radiographs before participating in any high-risk activity. Moreover, athletes are likely to suffer unstable fractures and dislocations in the cervical spine. Most of these fractures occur in the lower cervical spine [56]. The anterior column shortens when an athlete suffers a compressive flexion injury due to axial force and bending moments. Donnally III et al. and Heary et al. highlight that when this injury is completely compressive, a burst fracture may be caused due to axial loading [57,58], resulting in facet dislocation due to flexion-distraction injury

The primary purpose of this review is to shed light on the wide range of spinal injuries occurring in young athletes who participate in various sporting activities. The review will also illustrate the mechanisms of spine injuries that contribute to adverse outcomes in different sporting activities. Additionally, the review will demonstrate the importance of radiologists’ review in diagnosing and managing various spine injuries in each sporting activity.

## Review

Methods

Study Design

A multi-process design was used to review, evaluate, and demonstrate the wide range of spinal injuries in young athletes participating in different sporting activities. This paper also sought to demonstrate the importance of radiologists’ review in diagnosing and managing different spine injuries in each sporting activity by employing a systematic review design.

Information Sources and Reporting

Five electronic databases were searched under a priori protocol from PROSPERO and the Preferred Reporting Items for Systematic Reviews and Meta-analysis (PRISMA) guidelines [15]. The databases searched were as follows: Cochrane Library, PubMed, EMBASE, Web of Science, and Google Scholar. For an effective search, different keywords and the Boolean operators ‘AND’ and ‘OR’ were used. The keywords used were as follows; (Spine OR spinal cord OR cervical spine OR lumbar spine) AND (Injuries OR damages) AND (Sports OR Sporting activities OR Athletes) AND (Radiology OR Study). Moreover, the references from the included studies were scoured for additional related studies.

Eligibility Criteria and Guidelines

The inclusion and exclusion criteria were used to find relevant and original studies that evaluated spine injuries in sports. The inclusion criteria were as follows: (1) studies that were conducted in the English language; (2) studies that evaluated any form of spine injuries in athletes or any sporting activity; and (3) studies that evaluated treatment, management, or diagnosis of spine injuries in athletes. The exclusion criteria were as follows: (1) studies focusing on spine injuries in non-athletes and (2) studies published in a language other than English since the accurate translation of scientific terms could often be challenging. Retrospective studies involving participants were mostly included in the meta-analysis.

Search Strategy

**Figure 1:**
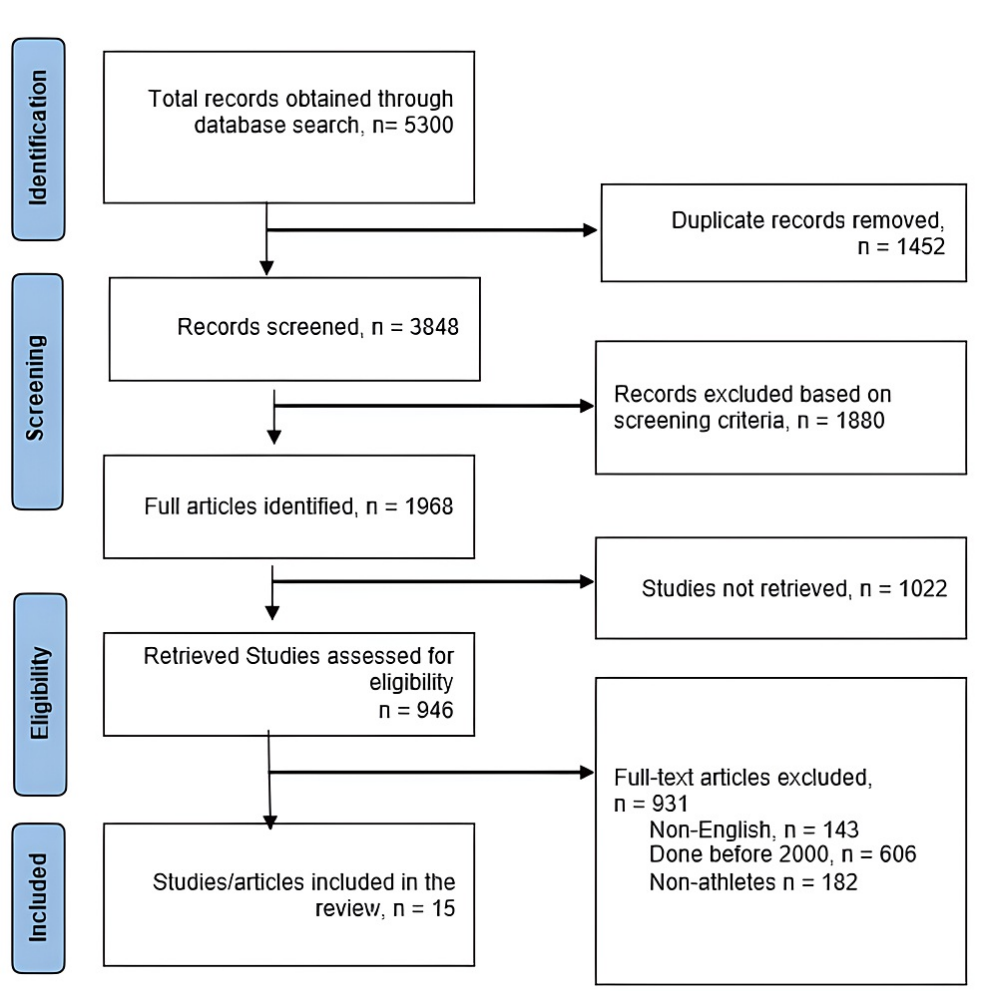
PRISMA flow chart illustrating the search strategy and inclusion of studies PRISMA: Preferred Reporting Items for Systematic Reviews and Meta-analysis

To identify published studies, a literature search was conducted on five databases - spanning the period between 2000 and 2022. The figure below depicts the search strategy based on the PRISMA guidelines (Figure 1). The search terms were modified for each database. Moreover, reference lists from the included studies were searched to enhance the database literature search. Abstracts and titles of potentially relevant published studies were screened for eligibility. The full texts of the articles and studies that met the inclusion and exclusion criteria were obtained and included in the systematic review.

Data Extraction

Two reviewers screened the articles based on the inclusion and exclusion criteria. Articles that satisfied the inclusion criteria were critically analyzed, and data were retrieved as per the PICO guidelines. The data retrieved from these articles included author, year of publication, study design, sample size (number of participants), sporting activities involved, type of spine injury, and outcomes. All the data from the studies that met the inclusion criteria were extracted and inserted into a table containing the above-specified variables. During the data extraction process, any inconsistencies were resolved by consultation with a third reviewer.

Quality Assessment and Data Synthesis

Through the Cochrane Review Manager Software, the Cochrane risk-of-bias tool was applied for statistical analysis of the selected 10 retrospective studies. The tool was significant in analyzing both the risks of bias and the quality of study articles. The selection of 10 retrospective studies was critical to estimate pooled results using the meta-analysis when studies evaluate spinal injuries and radiographic outcomes. Continuous data were pooled using the inverse variance method and dichotomous data using the Mantel-Haenszel method. For the five studies for which a meta-analysis was not possible, we offer a narrative summary of the results from individual studies.

Results

The initial search for articles evaluating spine injuries in sports activities and their management, treatment, and diagnosis elicited 5300 relevant records (Figure 1). When the reviewers screened the articles for duplicates, 1452 articles were eliminated. Using the inclusion and exclusion criteria, out of the 946 studies, 931 articles were excluded; 143 studies were found to be in languages other than English, 606 were conducted before 2000, and 182 studies involved spine injuries in non-athlete patients.

Study Characteristics

The characteristics of the included studies are presented in Table 1.

**Table 1:** Characteristics of included studies

Study	Study design	Participants	Sporting activities	Type of spine injuries	Outcomes
Pirruccio et al. (2020) [59]	Cross-sectional retrospective epidemiological study	Pediatric patients aged below 18 years	Football, bicycling, snowboarding, horseback riding, swimming and diving, snow skiing, skating, soccer, cheerleading, hockey	Acute vertebral fractures	Lumbosacral fractures were the most prevalent injuries, while coccygeal fractures were the least. Acute vertebral fractures were more prevalent in adolescent patients between the ages of 13 and 15 years
Plais et al. (2019) [60]	Epidemiology, descriptive research	-	Soccer	Lumbar, thoracolumbar, and cervical spine injuries	Lumbar disc herniation is diagnosed using an MRI. Thoracic disk herniation is rare and only represents about 0.25% to 0.75% of all disc herniations. Young athletes are at a high risk of spondylolysis and spondylolisthesis. Spondylolysis and spondylolisthesis are diagnosed using radiographic assessment such as MRI, CT scans, and X-rays
McNally et al. (2005) [48]	Descriptive research	-	Rowing	Annular tears, disc, and facet degeneration. Spondylolysis	Low-back pain problems are common in rowers. Rowers who habitually row on one side are at high risk of back pain problems related to asymmetric development. Spondylolysis is more common in rowers compared to other sporting activities. Imaging techniques used in diagnosing spondylolysis are MRI, CT scans, and SPECT. CT scans provide the best depiction of the bone anatomy of the par. MRI is essential in differentiating normal, acute, or chronic fractures
Tator et al. (2004) [61]	Retrospective study	Canadian athletes aged between 11 and 50 years	Ice hockey	Spinal injuries in general	Spine injuries were more common in young athletes aged between 16 and 20 years (49.0%). The main cause of spinal injuries was pushing/checking from behind, accounting for about 36.6%. Of the 256 cases reported, 83.5% involved cervical vertebrae, 5.1% thoracic vertebrae, 5.9% thoracolumbar, and 5.5% lumbosacral
Schmitt et al. (2001) [62]	Clinical and radiological study	21 male athletes with a mean age of 20 years	Javelin	Lumbar spine injuries	The most common lumbar spine injuries in javelin throwers are spondylolysis and spondylolisthesis. Nine athletes with spondylolisthesis and two with spondylolysis were associated with lumbar pains. Radiographs were essential in the demonstration of spondylolisthesis in athletes
Dunn (2010) [63]	Clinical study	27 male patients with a mean age of 25.3 years	Rugby	Cervical spine injuries	Most cervical spine injuries occur during a scrum (accounting for 37%). The player position highly associated with cervical spine injuries is scrumhalf, accounting for about 22% of the injuries. The injury distribution was as follows; 37% for unifacet dislocation, 22% for bifacet dislocation, 11% for dens type II fracture, and 5% for SCIWORA (spinal cord injury without radiological abnormality)
Baranto et al. (2009) [64]	Follow-up study	71 male athletes with a mean age of 26 years and 21 non-athletes	Weightlifting, wrestling, ice hockey, and orienteering	Thoracolumbar spine injuries	High spine abnormalities are observed in wrestlers and weightlifters because of heavy loading. Disc degeneration spine abnormalities are highly observed in ice hockey players. MRI was essential in the diagnosis of spine injuries as well as for showing the changes in spine abnormalities
Hubbard et al. (2011) [65]	Retrospective study	8634 patients with a mean age of 33.3 years	Snowboarding and skiing	General spinal injuries	Skiers had higher rates of cervical spine fracture or dislocation (3%), followed by thoracic and lumbar spine injuries (2.7% and 2.5%, respectively). Snowboarders were at higher risk of lumbar or thoracic spine injuries than cervical spine injuries
Schmitt et al. (2004) [66]	Radiological study	159 German male athletes with a mean age of 45.8 years	Field and track sports	Lumbar spine injuries	Osteophytes in the lumbar spine were more prevalent in shot put or discus throwers compared to the other disciplines
Nyska et al. (2000) [67]	Clinical Study	4 young athletes	Swimming	Spondylolysis	Routine X-ray examinations are essential for the diagnosis of spondylolysis and spondylolisthesis. The most common symptom exhibited by the patients was pain in the lower back area. The pain was exacerbated by hypertension, side flexion to the injured side, and extreme flexion
Yokoe et al. (2020) [68]	Retrospective comparative study	267 young athletes aged between 7 and 18 years with lower back pain	Soccer and baseball	Spondylolysis	MRI scans were essential in diagnosing spondylolysis as the cause of lower back pain in the patients. MRI scans showed that 56 lesions were observed in 33 soccer players while 60 lesions were observed in baseball players. 55.4% of soccer players and 60% of baseball players showed high signal-intensity lesions
Browne (2006) [69]	Retrospective descriptive case series study	Children under the age of 15 years	Rugby	Cervical spine injury	Most cervical spine injuries occurred in boys over 11 years of age (accounting for 97% of the injuries). The results showed no association between player position and cervical spine injuries
Shelly et al. (2006) [47]	Retrospective study	12 Irish athletes with a mean age of 21.6 years	Rugby	Cervical spine injury and thoracic spine injury	Hyperflexion was the most common injury mechanism as it occurred in nine patients, while for three patients, hyperextension was the mechanism of the injury. Most of the injured players were playing as fullbacks, hookers, or wings
Kim et al. (2016) [70]	Retrospective study	275 children with a mean age of 12 + 4.5 years	Wide range of sports	Spinal injuries in general	Hockey accounted for most of the sport-related spine injuries. MRI images are used to reveal contusions or hematomas of the spinal cord. The age group most associated with spine injuries among sport-related causes is between 14 and 18 years
Ellis et al. (2019) [71]	Retrospective cohort study	266 patients with suspected sport-related concussions aged between 6 and 19 years	Hockey, football, soccer, and basketball	Cervical spine injury	CT scans and MRI diagnosing imaging modalities were used to check for abnormalities in the cervical spine among athletes. Hockey accounted for the highest cervical spine dysfunction for patients with SCR (accounting for 50% of the injuries)

Meta-analysis

Plots

**Figure 2:**
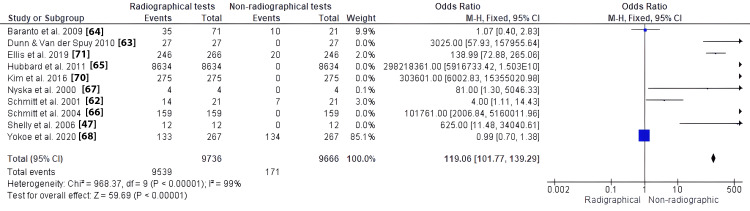
Forest plot for comparison 2. Radiographical review versus non-radiographical review in identifying and minimizing risks of spinal injuries among athletes; outcome: 2.1. radiographical and non-radiographical tests of spinal injuries

**Figure 3:**
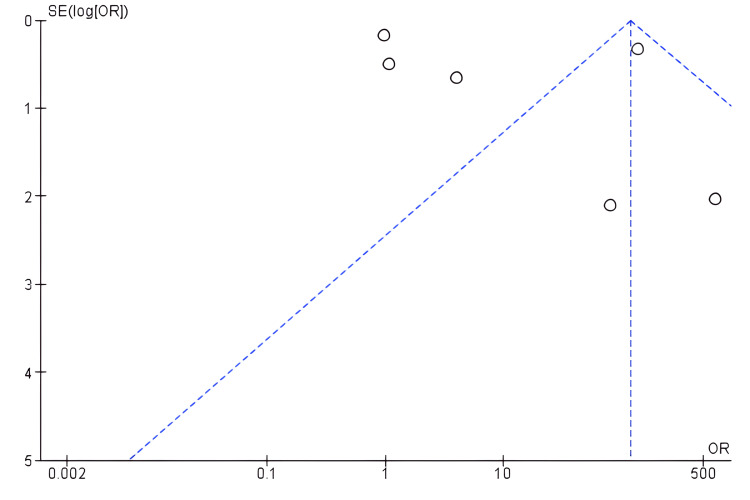
A funnel plot of radiographical tests in identifying spinal injuries versus non-utilization of radiographical tests in identifying spinal injuries among athletes

A meta-analysis was conducted on 10 out of 15 included studies to evaluate the significance of radiographical tests in discovering spinal injuries among athletes. Ten studies were selected since they were retrospective in design. Figure 2 shows a forest plot illustrating the radiographical review of spinal injuries among athletes. Radiographical review - such as MRI and CT scan - improves the chances of evaluating spinal injuries (OR: 41.33, 95% CI: 37.08 to 46.06). Figure 3 presents a funnel plot illustrating the significance of the radiographic review of spinal injuries since its utilization identifies high incidences of spinal injuries.

The plots of the studies evaluated radiographical tests in terms of identifying spinal injuries among athletes. Its utilization effectively identifies the injuries, making it effective for professionals to manage them among athletes. Studies that rarely used radiographical tests hardly detected spinal injuries among athletes. In testing heterogeneity among the included studies for meta-analysis, inverse variance (I^2^) was used to demonstrate the level of discrepancy. According to Higgins, I^2^ statistics of 25%, 50%, and 75% correspond to low, moderate, and high heterogeneity, respectively [72]. Based on the Review Manager analysis (Figure 2), the included studies demonstrated substantial heterogeneity (I^2^ = 99%, p = 0.00001), indicating the utilization of prediction for interpretation and computing random effect model meta-analysis.

Risk of Bias

**Figure 4:**
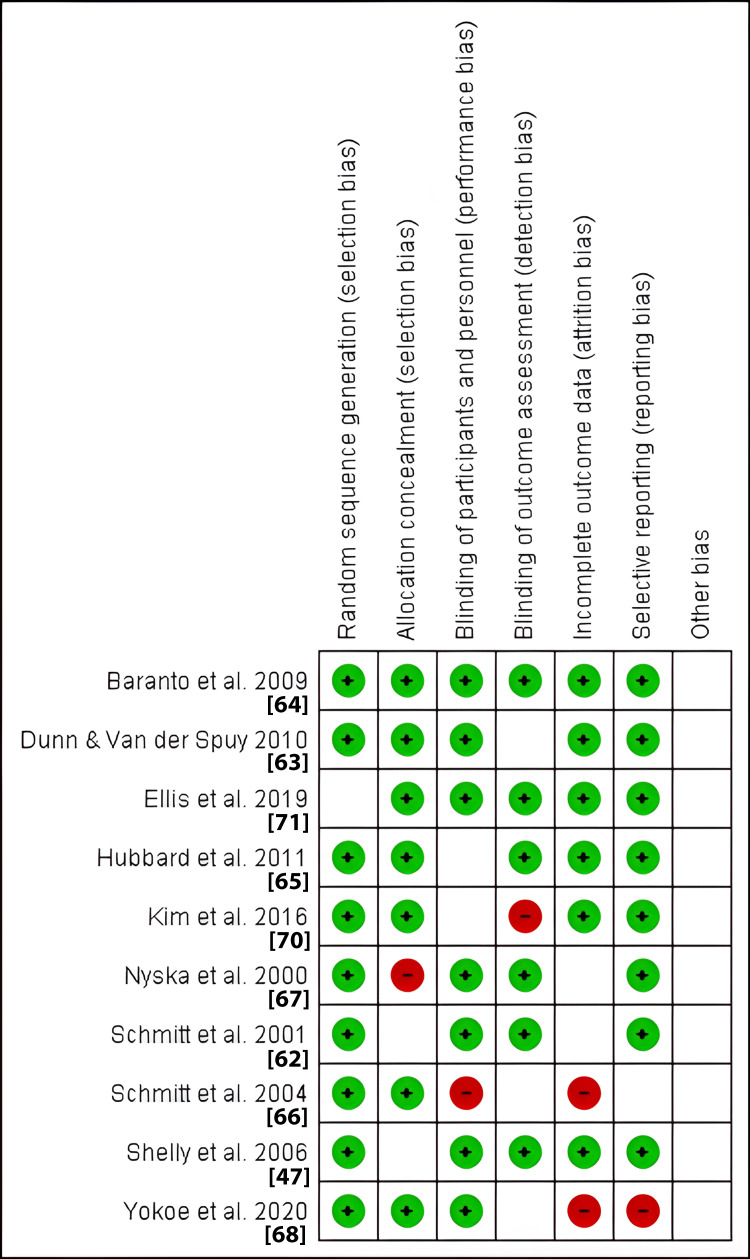
Risk of bias summary The image summarizes the review authors’ judgments about each risk of bias item for each included study. A green circle illustrates a low risk and a red circle represents a high risk. The absence of both represents an unclear risk of bias

**Figure 5:**
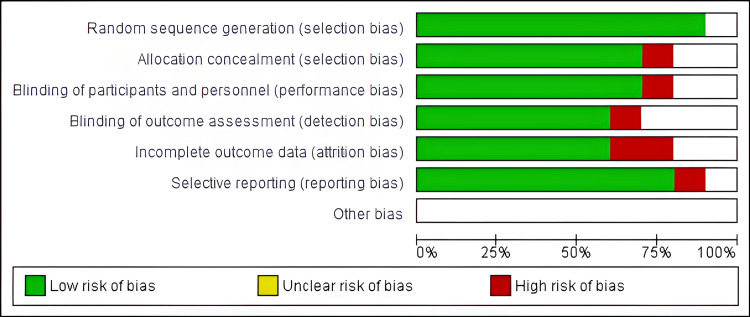
Risk of bias graph The review authors’ judgments about each risk of bias item presented as percentages across all included studies

The Review Manager Software was also significant in evaluating the risk of bias in included studies under meta-analysis. The items evaluated were as follows: allocation (selection bias), blinding (detection and performance bias), attrition bias, selective reporting bias, and other potential sources bias. Figures 4 and 5 below provide a summary of the risk of bias and risk of bias graph, respectively.

Narrative Summary of the Results

The included studies have revealed that some patients who experience low-back pain after an MRI or CT scanning are diagnosed with lumbar bone fractures. These fractures are usually seen in athletes involved in sporting activities that require repetitive lumbar extensions and rotation, such as gymnastics, wrestling, weightlifting, and baseball. We have also established that stingers are observed in patients engaged in contact or collision sports such as football, rugby, and hockey. This injury is very common in sports but is least understood by most; most athletes tend not to report it as they consider its effects insignificant. The mechanisms of this injury are traction and compression. The traction injury always occurs in the branchial plexus when the neck is flexed laterally, and the shoulder is contralaterally flexed. Axial loading has also been found to lead to spine injuries. For most of the footballing patients who suffer from permanent quadriplegia, the involved injury mechanism was axial loading.

Similarly, patients in skiing and snowballing have been found to suffer from spine injuries due to failed jumps and axial loading. On the other hand, catastrophic cervical spine injuries in diving, gymnastics, rugby, and ice hockey have been attributed to axial loading. Axial loading in athletes is usually brought about by intentionally using the crown of the head as a point of contact. Ice hockey is highly associated with disc degeneration; this may be due to the combination of high-speed skating with forceful tackles that occur in off-balance positions. Another study has shown that Schmorl’s nodes are present in athletes in orienteering. This injury was attributed to the differences in spine loading in orienteering compared to vigorous activities such as wrestling and weightlifting.

The distribution of spondylolysis lesions on MRI in baseball and soccer players differs, with soccer players showing more multiple lesions compared to baseball players. The study attributed this increase in multiple lesions in soccer players to specific movements such as jogging and running during training without kicking a ball. However, spondylolysis has been described as a stress-related injury and is found mostly in athletes who exert extreme loads on the lumbar spine. In swimmers, the ones who emphasize the breaststroke and/or butterfly style are at high risk of spondylolysis because these styles involve repeated flexion and hyperextension of the lumbar spine.

When it comes to the diagnosis of spondylolysis, CT scanning has been shown to demonstrate the lesions more accurately. However, SPECT is a powerful tool for bone scanning and differentiating spondylolysis from spondylolisthesis. Cervical spine injuries are common in athletes engaged in collision sports such as football. These injuries are usually attributed to neck extensions, flexions, and rotation forces. In skiing athletes, there is a higher risk of cervical spine dislocation or fractures. These injuries were attributed to falls or collisions. On the other hand, snowboarding athletes are more likely to suffer from lumbar or thoracic spine injuries. These injuries are attributed to intentional jumps that lead to landing on the buttocks or with increased force on the legs.

Discussion

The number of adults and adolescents involved in sporting activities is significantly on the rise. Sport-related spine injuries are rare but not uncommon. Several studies have been conducted to show the relationship between different sporting activities and spine injuries. The sport-related spine injuries range from lower back pains to catastrophic cervical, thoracic, and lumbar spine injuries. Spine injuries in sports are attributed to certain common injury mechanisms, such as axial loading, hyperextension, hyperflexion, traction or compression of the branchial plexus, repetitive axial loading, eccentric muscle contraction, or multiple repetitive traumatic episodes [73]. Axial loading results in rotational forces that lead to dislocations [74]. Similar to axial loading, hyperflexion injuries involve a certain degree of rotation [75].

Instability caused by hyperflexion injuries is usually associated with the disruption of the posterior longitudinal ligament. A combination of hyperflexion and rotation force can be catastrophic as they can disrupt the integrity of supportive soft tissues. In adolescent athletes, hyperflexion usually affects the C5-6 region. On the other hand, hyperextension is usually associated with falls. In cases of violent hyperextension, the spinal cord can be damaged and cause a pincer mechanism. Traction injuries usually affect the vascular connective tissues in the cervical nerve roots. The compressive and traction forces on spinal nerve roots are usually a result of lateral bending. Excessive traction on the peripheral nerves results in neurological conditions such as stingers. Additionally, excessive lateral bending can place significant compressive and tractional forces on the spinal nerve roots as well as on the sheath of the dorsal nerve root [76,77]. These injury mechanisms related to some of the sporting activities are summarized in Table 2.

**Table 2:** Sporting activities and mechanisms of injury

Sporting activity	Mechanisms of injury
Football	Hyperflexion and axial loading
Ice hockey	Axial forces
Rugby	Flexion, extension, distraction, rotation, hyperflexion, and hyperextension
Skiing and snowboarding	Jumps, falls, chairlifts, and collisions
Soccer	Flexion/extension, hyperextension, hyperflexion, and repetitive rotational forces
Basketball	Combination of torsion, loading, and trauma

Football

In the United States, Football is a popular sport, with many individuals participating in the sport from the early teenage onwards (15-18 years) [78]. The most common spine injury incurred by most football athletes occurs on the cervical spine. In this review, two studies have evaluated cervical spine injuries in football. One study reported that with a 95% confidence interval, football accounted for 23.4% of cervical spine injuries. Similarly, a study conducted by Ellis et al. showed that out of 80 patients diagnosed with a sport-related concussion and cervical spine dysfunction, football accounted for about 6% [71]. However, previous studies have shown that lumbar spine injuries are also common, i.e., 76% of disc herniation in Football athletes occurred in the lumbar spine and were mostly located in the L5-S1 disc [79]. Another common spine injury experienced by football patients is spondylolysis, attributed to extension and rotation forces in the lumbar spine. Football is also associated with catastrophic cervical injuries, which mostly occur in the C5-C6 level. These catastrophic injuries are associated with axial forces exerted on top of the head when the neck is slightly flexed. A previous study has reported that football is also associated with cervical cord neuropraxia, with a prevalence of 7 out of 10,000 football participants [80]. The mechanism associated with this injury is hypothesized to be either hyperflexion or hyperextension of the neck, which causes pincer-type compression injury to the spinal cord.

Ice Hockey

This sport is common in the United States and Canada. Several studies have been conducted on the prevalence of spine injuries and mechanisms in the sport. In this review, ice hockey's relationship to spine injuries has been discussed in four studies. The study by Pirrucio et al. reported that hockey (including ice hockey) accounted for 2.5% of acute vertebral fractures [59]. Another study by Kim et al. has shown that hockey accounted for most of the spine-related injuries, i.e., out of 51 sport-related spine injuries, 17 were linked to hockey [70]. In this sport, the most common spine injuries occur in the cervical area. These injuries occur due to different mechanisms, with pushing/checking from behind the most common injury mechanism. According to a study by Tator et al., pushing/checking accounted for 36.6% of the injuries [61].

The incidence of other injury mechanisms was as follows: 23.7% pushed/checked, 19.8% tripped on ice, 8.9% slid, and 3.5% tripped by another player. Collisions are also known to cause spine injuries in hockey players. Collision with boards accounts for most of the injuries, with a frequency of about 65.8%. Previous studies seem to support this hypothesis. For example, according to Mölsä et al., checking from behind and head collision with boards accounted for 50% of the injury mechanisms [81]. These collisions to the head subject the neck to axial loading, resulting in cervical spine injuries. Additionally, slight flexion of the neck after a collision with the board increases the risk of cervical fractures. Despite the use of helmets as protective gear in this sport, studies have shown that the helmets are incapable of sufficiently minimizing axial loading, especially during spear tackling [82,83]. It is therefore recommended that officials enforce strict regulations on head-down hitting rules.

Rugby

Rugby is a very popular sport worldwide. The aggressive style of play associated with this sport is linked to a high risk of spine injuries, especially cervical spine injuries. A study involving athletes below 19 years reported that low spine-related cases were observed in rugby [70]. This can be attributed to the low number of adolescents involved in this sport. Similarly, in previous studies conducted by Scher et al. over 10 years (1987-1996), a total of 87 players were diagnosed with spinal cord injuries [84]. Scrum and tackling plays are associated with high cervical spine injuries. According to a study by Dunn et al., the common plays associated with most cervical spine injuries were tackling and scrum with a frequency of 52% and 37%, respectively [63]. On the other hand, Maul and ruck play contributed 7% of the injuries.

A study by Browne (2006) among young rugby athletes reported that hyperextension injury mechanisms accounted for most of the cervical spine injuries (30%) [69]. Frequencies of the other injury mechanisms were as follows: 7% extension, 13% flexion/extension, 6% distraction, 2% rotation, and 30% flexion. Similarly, another study involving 12 Irish rugby players revealed that nine of the players suffered from spine injuries due to hyperflexion while hyperextension was observed in three players [47]. Most flexion injuries result from scrum play. For instance, in the study by Dunn et al., 23% of flexion injuries occurred during scrum play [33]. On the other hand, rotation/hyperextension injuries are common during spear tackles.

Soccer

Soccer is regarded as the world’s most popular sport. The common spine-related injuries in this sport are low-back injuries. Soccer players are at risk of spondylolysis. which is the main cause of low-back pain in young athletes [68]. The cause of this injury among soccer players is due to lumbar flexion-extension movements, rotational forces, and lumbar compression forces that mostly occur during training exercises that involve no kicking of the ball. Another study by Plais et al. claimed that soccer players are at risk of overuse and acute spine injuries due to hyperflexion, kicking, heading, hyperextension, and repetitive rotational movements associated with the sport [60]. Studies have also shown that soccer players are likely to suffer from other lumbar spine injuries such as sprains. A study involving 310 lumbar spine injuries among soccer players revealed that 49.4% were associated with low-back pain, 15.2% were sprains, and spondylolysis accounted for 4.2% [85].

Skiing and Snowboarding

According to Xiang et al., the most common injuries among severely injured skiers and snowboarders are traumatic brain injuries and vertebral injuries [86]. Several studies have been conducted to show the relationship between spine injuries and skiing and snowboarding. One study by Hubbard et al. reported that skiers had higher rates of fractures or dislocations in the cervical spine [65]. In snowboarders, the lumbar and thoracic spine were more susceptible to injuries. The most common attributed mechanism of these injuries is falling. However, a previous study by Tarazi et al. reported that for skiers and snowboarders, the most common mechanism of injury was jumps, which accounted for 77% of the injuries [87]. Other mechanisms of injuries such as falls and collisions accounted for 18% and 5%, respectively. The study showed that no spine injuries are related to chairlifts. Another study also reported that in skiers and snowboarders, lumbar injuries were common, i.e., 64.8% of skiers suffered lumbar injuries while 69.45% of snowboarders suffered lumbar injuries. The spinal injuries commonly associated with snowboarders were attributed to simple falls and intermediate or expert jumps.

Basketball

Basketball is one of the major global sports with approximately 450 million players worldwide [88]. The sport's physical nature and intensive movements make the players vulnerable to several injuries. However, this sport accounts for very few spine-related injuries. For example, according to one of the studies included in this review, out of 80 athletes with sport-related concussion and cervical spine dysfunction, basketball only accounted for 7.5% [71]. Similarly, another study by Kim et al. reported that basketball was among the sports that contributed least to spine-related injuries among young athletes [70]. However, a previous study by Drakos et al. on the overall injuries experienced by basketball players in the NBA reported that lumbar spine injuries accounted for 10.2% of all injuries [89]. The lumbar spine injuries were second after ankle injuries, which accounted for 14.7% [89]. The study also shows that out of the 10.7% of lumbar spine injuries, sprain and strains accounted for 7.9%, lumbar disc degeneration accounted for 0.9% and lumbosacral degeneration accounted for 0.9% [89]. According to Ball et al., the lumbar spine injury mechanism is likely related to a combination of torsion, loading, and trauma [78].

Importance of Radiographical Assessment in the Diagnosis and Management of Spine Injuries

The purpose of radiological assessment is to help document lesions that must be treated, such as disc herniation and instability [90]. For example, according to Schmitt et al. (2004), radiographs performed on javelin athletes revealed spondylolisthesis in 10 athletes, whereby in eight of the athletes, they were in L5/S1, and at L4/L5 in two athletes [66]. Additionally, the radiographs were able to show whether there were any abnormalities in the par interarticular. MRI images of a 29-year-old ice hockey player in a study by Baranto et al. revealed Schmorl’s node in the lower endplate of Th11 and normal disc [64]. These were essential in determining the treatment the individual was supposed to undergo. Additionally, imaging tests are usually important in determining the potentially serious lesions in athletes who show signs and symptoms of instability or neurological deficits.

To decrease the chances of missing fractures, additional views are needed. Most of the included studies discuss radiological assessment usually beginning with plain radiographs and frontal, lateral, and odontoid projects. Plain radiographs also remain useful in detecting mild spine trauma and demonstrating fractures, especially those near the cervicothoracic junction. However, according to McNally et al., imaging evaluations should always start with MRI [48]. The study states that MRI has no ionizing radiation and provides the best overview of potential causes of back pain in athletes. Additionally, CT usually provides the best depiction when it comes to bone anatomy. Studies over the years have shown that CT scans can document cervical fractures that are difficult or impossible to see using plain radiographs [91-93]. In the past, the gantry was angled along the long axis of the pars. However, this is not necessary for newer helical or multislice equipment, and high-resolution imaging in the axial plane supported by sagittal reconstructions depicts pars anatomy clearly [48].

Limitations

This study reviewed results from various literature sources and hence was bound to have certain limitations. The primary limitation of our study is that it only considered documents written in English, meaning that relevant information in other languages that would have contributed to this study was omitted. The other limitation is that some of the included studies had a small sample size, which affects the generalizability of our findings to a broader population. The review also considered descriptive epidemiological studies, which reported results obtained from previous research, thus complicating our research process. The descriptive research studies also did not provide any radiographical images, posing a challenge in terms of identifying the right radiographic assessments that are used to diagnose different sport-related spine injuries. The limitation of the meta-analysis involved our decision to use only retrospective studies from the included ones. Consequently, the meta-analysis used only 10 retrospective studies despite including 15.

Implications

The main purpose of this study was to evaluate the common spine injuries in athletes in various sports and assess their injury mechanisms. We have provided a comprehensive assessment of spine injuries in different sports such as football, soccer, rugby, ice hockey, skiing, and snowboarding. We hope that our findings will enable radiologists to obtain the right diagnostic tests and thereby appropriately guide the management or treatment of the injury. Additionally, the study has shown that the injury in athletes usually depends on different mechanisms and loads on the spine relating to specific patterns of injuries. This knowledge is beneficial for both athletes and the sports administrations as it can help them adopt the right protective attires to help prevent or minimize injuries. This study is exploratory and interpretative in design, and it lays a strong foundation for future research in both theory development and concept validation.

## Conclusions

Spine injuries are common in elite athletes in several sports. About 10% of spinal injuries in the United States result from sporting activities. In diagnosing spine injuries, imaging tests such as MRI, CT, or plain radiographs are essential. From a radiologist's perspective, these tests are highly valuable as they can help decide which treatment is required for a particular athlete or how the injury can be managed. Recovery from a specific spine injury usually depends on the kind of injury and the rehabilitation process the athletes undergo before returning to play.
